# Feasibility of Mediastinal Radiation Use in HeartMate 3 LVAD Recipients with Cancer

**DOI:** 10.14797/mdcvj.1115

**Published:** 2023-01-23

**Authors:** Nausharwan Butt, Farooq H. Sheikh

**Affiliations:** 1 Einstein Medical Center, Philadelphia, Pennsylvania, US; 2 MedStar Heart and Vascular Institute/Georgetown University, Washington, DC, US

**Keywords:** HeartMate LVAD, mediastinal cancer, radiation exposure, stereotactic bone radiation therapy

## Abstract

The HeartMate 3 left ventricular assist device possesses the unique feature of having its electronics and software located within the housing of the pump, which may predispose it to malfunction from radiation exposure during cancer treatment. We investigated this association in a case series of two patients at our institution.

## Introduction

The HeartMate left ventricular assist device (HM3 LVAD, Abbott) achieves mechanical rotation and circular levitation by uninterrupted electromagnetic support.^1^ A unique aspect of the HM3 device is that the electronics and software required to operate the pump are stored within the housing of the pump. This structural aspect raises concern regarding the safety of radiation use in LVAD patients with mediastinal cancers. Literature on radiation therapy in LVAD patients is limited, with initial studies suggesting no significant complications in older axial flow pumps.^2,3,4^ However, the effect of radiation on HM3 LVAD performance is not well known. We report a case series of stereotactic bone radiation therapy (SBRT) in advanced heart failure patients with cancer who are supported by an HM3 LVAD implantation.

## Case Series

### Patient 1

**Figure 1:**
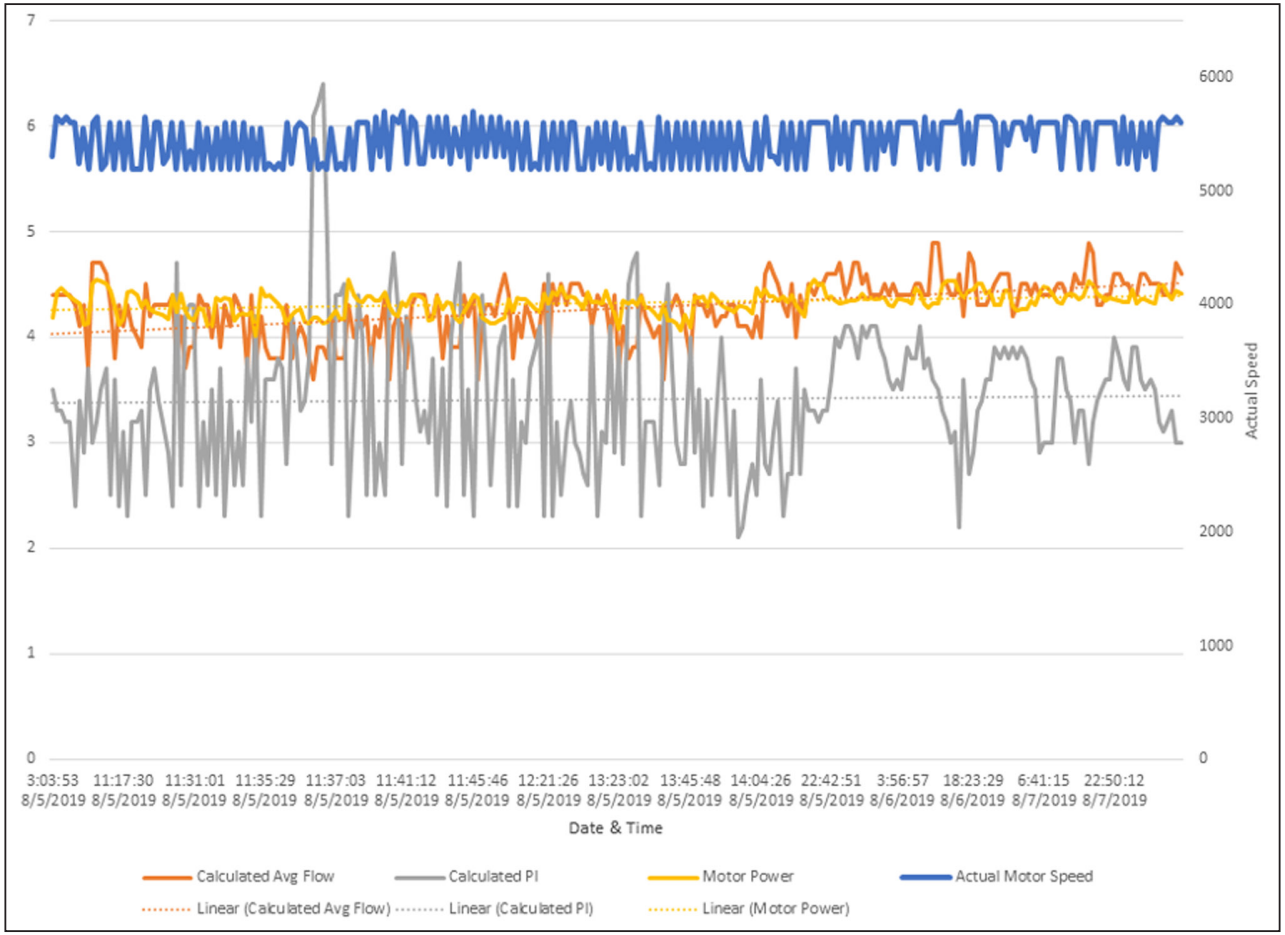
Left ventricular assist device event logs for patient 1 during first radiation treatment. Reprinted with permission. Blue line: motor speed (RPM); orange line: calculated average flow (L/min); yellow line: motor power (watts); grey line: calculated pulsatility index

A 71-year-old woman had a history of advanced systolic heart failure status post HM 3 LVAD and left upper-lobe non-small cell lung cancer status post lobectomy. She was found to have an incidental left-sided chest wall mass on computed tomography (CT) imaging, with subsequent biopsy demonstrating oligometastatic lung adenocarcinoma. She consequently underwent 5 fractions of 600 centigray (cGy) SBRT to her left lateral chest wall over a period of 10 days. Mild fatigue was reported following radiation; however, LVAD log files sent after each fraction were unremarkable (Figure 1, Table 1). During the first year of follow-up, device malfunction has not been witnessed.

**Table 1:** Left ventricular assist device event logs for patient 1 during first and fifth radiation treatment. RPM: revolutions per minute; W: watts


	FIRST TREATMENT	FIFTH TREATMENT

Pump speed (RPM)	5600	5600

Low speed limit (RPM)	5200	5200

Average pump speed (RPM)	5600	5473

Motor power maximum (W)	4.5	4.6

Motor power minimum (W)	4.0	4.0

Motor power average (W)	4.3	4.3

Flow maximum (Liters/minute)	4.9	5.1

Flow minimum (Liters/minute)	3.6	3.6

Flow average (Liters/minute)	4.3	4.4

Pulsatility Index maximum	6.4	5.9

Pulsatility Index minimum	2.1	1.9

Pulsatility Index average	3.4	3.6

Hematocrit (%)	35	35


### Patient 2

**Figure 2:**
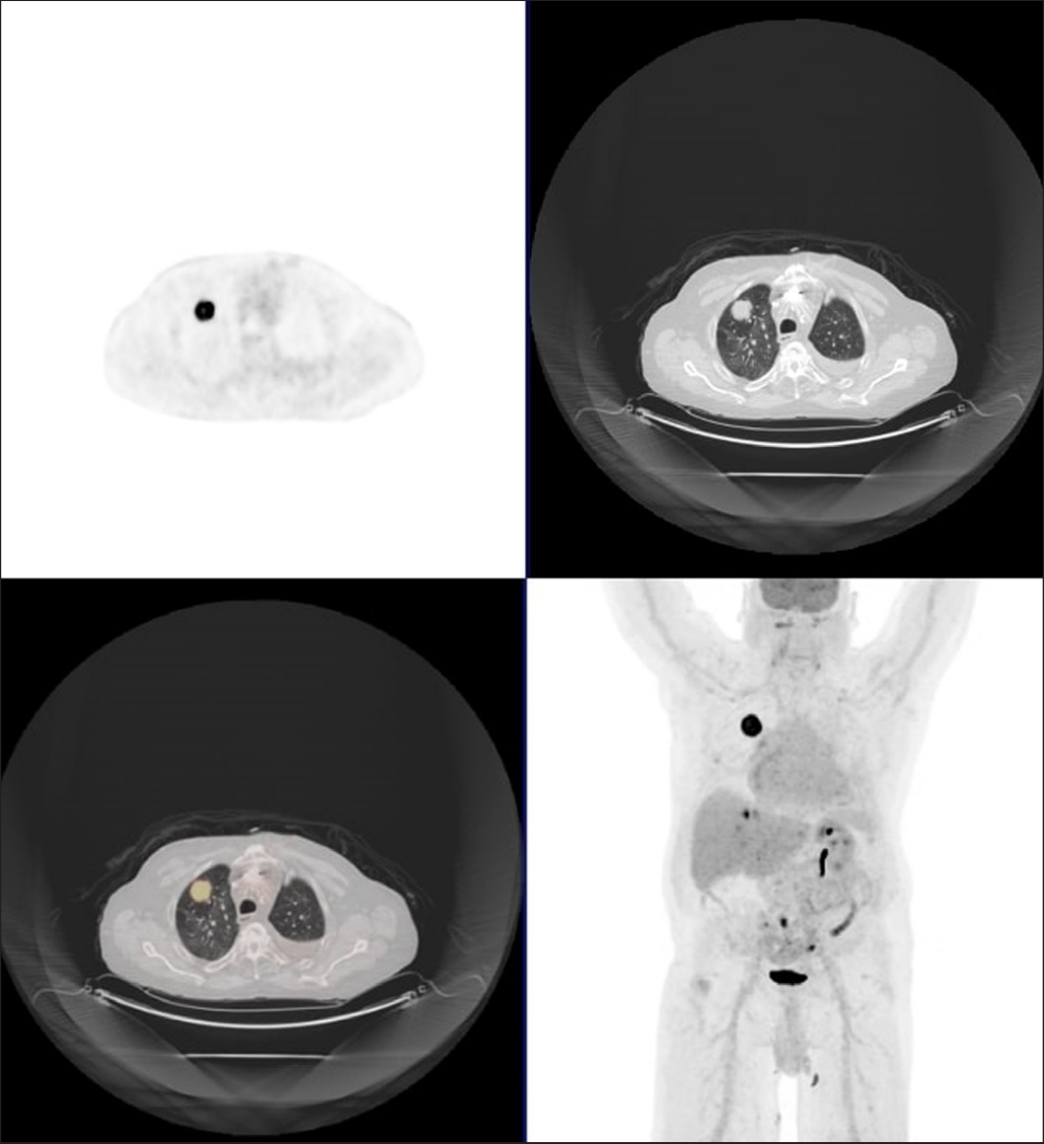
Fluorodeoxyglucose positron emission tomography SPECT imaging of demonstrating right upper lobe tumor (patient 2). SPECT: single photon emission computed tomography.

**Figure 3:**
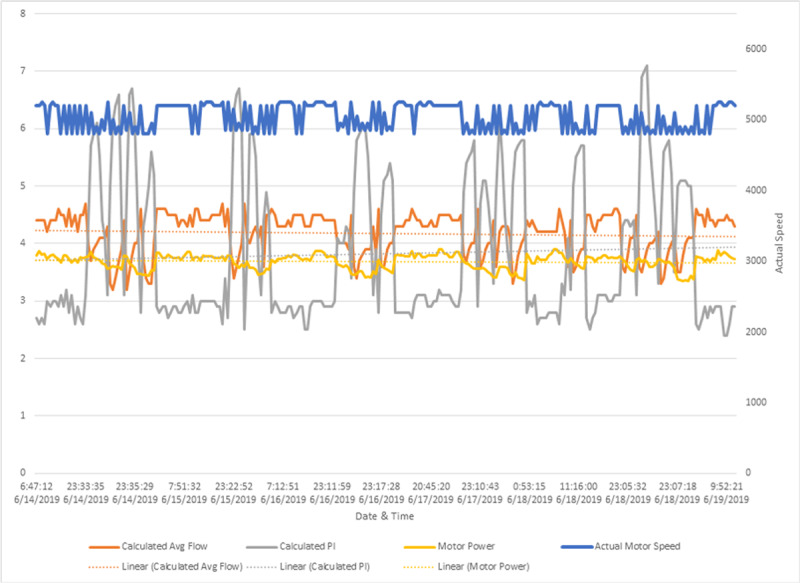
Left ventricular assist device event logs for patient 2 during first radiation treatment. Reprinted with permission. Blue line: motor speed (RPM); orange line: calculated average flow (L/min); yellow line: motor power (watts); grey line: calculated pulsatility index

A 70-year-old man with a history of advanced systolic heart failure (HF) and HM3 LVAD implantation presented with worsening dyspnea. He was found to have a right upper-lobe nodule with mediastinal lymphadenopathy on CT imaging (Figure 2). Ensuing biopsy diagnosed squamous cell lung cancer without metastatic disease. SBRT was initiated, and five treatments of 1,000 cGy were delivered to the right upper-lobe lesion within 10 days. No LVAD complications or symptoms of HF were witnessed during treatment or in subsequent follow-up (Figure 3, Table 2). On outpatient follow-up, the patient denied any symptoms and normal LVAD function was observed with flow 4.3 L/min, speed 5,200 rpm, pulse index 4.8, and power 3.8 watts.

**Table 2:** Left ventricular assist device event logs for patient 2 during first and fifth radiation treatment. PM: revolutions per minute; W: watts


	FIRST TREATMENT	FIFTH TREATMENT

Pump speed (RPM)	5200	5200

Low speed limit (RPM)	4800	4800

Average pump speed (RPM)	5200	5200

Motor power maximum (W)	3.9	3.9

Motor power minimum (W)	3.3	3.4

Motor power average (W)	3.7	3.7

Flow maximum (Liters/minute)	4.7	4.7

Flow minimum (Liters/minute)	3.2	3.2

Flow average (Liters/minute)	4.2	4.3

Pulsatility Index maximum	7.1	6.9

Pulsatility Index minimum	2.4	2.2

Pulsatility Index average	3.8	3.4

Hematocrit (%)	43	43


## Discussion

This case series demonstrates feasibility of mediastinal radiation in patients with HM3 LVAD implantation. Although uncommon, radiation treatment has been associated with malfunction of cardiac implantable electronic devices (CIEDs), which may result in loss of historical diagnostic data or resetting of the apparatus.^5^ This malfunction has primarily been associated with neutron-producing radiation, with a direct correlation between the approximate cumulative radiation dose at the CIED location and probability of an adverse event.^6^

Current data on radiation use in CIEDs remains largely limited to permanent pacemakers and implantable cardioverter defibrillators. There is limited published data regarding the safety of mediastinal radiation in LVAD recipients. The HM3 LVAD utilizes magnetic levitation to substantially reduce friction wear, with the essential electronic components required for continued operation uniquely located within the motor in the lower housing of the stator.^7^ This position directly exposes the electronic components during radiation therapy. Our two patients both received a substantial amount of radiation (3,000 and 5,000 cGy), although LVAD parameters remained within operational limits. Our findings concur with those of Spano and colleagues, who recently reported the first successful use of SBRT in a patient with HM3 LVAD implantation without device malfunction.^8^

As organ donation continues to be a finite resource, the use of LVADs continues to grow. The HM3 LVAD, the predominant LVAD therapy, essentially eliminates mechanical friction within the rotor, thereby reducing associated device failure and adverse events. Given the increasing long-term survival of LVAD patients, the treatment of non-cardiac causes of morbidity and mortality, particularly cancers, is increasing. Our present study highlights the feasibility of radiation therapy with the HM3 LVAD.

These case series findings must be viewed with some caution given that it is only comprised of two patients from a single institution within the US. Larger, multi-institutional studies are warranted to further evaluate the safety profile and the development of best practices for the use of mediastinal SBRT in patients with HM3 LVAD therapy.

## Conclusion

Mediastinal SBRT in cancer patients with an HM3 LVAD is feasible and appears to be well tolerated. Further studies are required to demonstrate safety and efficacy in this unique patient population.
